# The Golgi α-1,6 mannosyltransferase KlOch1p of *Kluyveromyces lactis *is required for Ca^2+^/calmodulin-based signaling and for proper mitochondrial functionality

**DOI:** 10.1186/1471-2121-10-86

**Published:** 2009-12-14

**Authors:** Elena Zanni, Francesca Farina, Antonella Ricci, Patrizia Mancini, Claudio Frank, Claudio Palleschi, Daniela Uccelletti

**Affiliations:** 1 Dpt. Developmental and Cell Biology, University LA SAPIENZA, P.le A. Moro,5 00185 Rome, Italy; 2 Pasteur Insitute-Fondazione Cenci Bolognetti, LA SAPIENZA, Rome, Italy; 3 Dpt. Experimental Medicine, University LA SAPIENZA, Rome, Italy; 4 National Centre for Rare Diseases, Istituto Superiore di Sanità, Rome, Italy; 5 Institut de Génétique et Microbiologie, UMR8621, Université Paris-Sud, 91405 Orsay Cedex, France

## Abstract

**Background:**

Protein *N*-glycosylation is a relevant metabolic pathway in eukaryotes and plays key roles in cell processes. In yeasts, outer chain branching is initiated in the Golgi apparatus by the alpha-1,6-mannosyltransferase Och1p.

**Results:**

Here we report that, in *Kluyveromyces lactis*, this glycosyltransferase is also required to maintain functional mitochondria and calcium homeostasis. Cells carrying a mutation in *KlOCH1 *gene showed altered mitochondrial morphology, increased accumulation of ROS and reduced expression of calcium signalling genes such as calmodulin and calcineurin. Intracellular calcium concentration was also reduced in the mutant cells with respect to the wild type counterparts.

Phenotypes that occur in cells lacking the alpha-1,6-mannosyltransferase, including oxidative stress and impaired mitochondria functionality, were suppressed by increased dosage of KlCmd1p. This, in turn, acts through the action of calcineurin.

**Conclusions:**

Proper functioning of the alpha-1,6-mannosyltransferase in the N-glycosylation pathway of *K. lactis *is required for maintaining normal calcium homeostasis; this is necessary for physiological mitochondria dynamics and functionality.

## Background

In eukaryotic cells, transmembrane and secreted proteins undergo several modifications during their maturation; *N*-Glycosylation is one of these modifications, and contributes to the functional conformation and to the selection of the final destination of these proteins [1]. Yeast and mammals share the initial steps of *N*-glycosylation in the ER, even if the pathways diverge between mammals and yeast in the Golgi apparatus. Indeed, in this cellular compartment, the extension of the oligosaccharide chain involves many specific glycosyltransferases. This specificity generates the observed diversity of glycan structures between different species and cell types [2]. In the yeast *Saccharomyces cerevisiae *the glycosyltransferases are constituted by mannosyltransferases, which lead to the formation of two main types of mannan outer chain. Many proteins of the cell wall and periplasm receive a large mannan structure that contains a long *α*-1,6-linked backbone of about 50 mannoses with short *α*-1,2 and *α*-1,3 side chains. In contrast, the proteins of the internal organelles display a smaller core-type structure, with only a few mannoses [3]. The structure of the mannan outer chain has been investigated by the study of *mnn *(mannan defective) mutants isolated by Ballou and co-workers [4]. The analysis of the partial *N*-glycan structures in these mutants has allowed the ordering of the steps of mannan synthesis. The work of Munro suggests a model for the complete *N*-glycosylation pathway in *S. cerevisiae *[3,5]. In this model, the formation of the mannan outer chain is initiated by the Och1p protein, a type II membrane *α*-1,6-mannosyltransferase that defines an early Golgi compartment [6-8]. Upon arrival in the Golgi, all *N*-glycan cores receive a single α-1,6-mannose added by Och1p. The formation of the long α-1,6-linked backbone is generated by two enzyme complexes: the M-Pol I and M-Pol II complexes.

*Kluyveromyces lactis *is a yeast species related to *S. cerevisiae*; its outer mannan chains differ from those of *S.cerevisiae *by having terminal N-acetylglucosamine and no mannose phosphate [9]. Previous studies from our laboratory reported that the *Kloch1-1 *mutation of *Kluyveromyces lactis *yeast was mapped at 738 bp of the *KlOCH1 *gene and we found that the corresponding wild type allele encodes the functional homologue of the *S. cerevisiae *α1,6-mannosyltransferase [10]. Quantitative analysis of cell-wall components indicated a noticeable increase of chitin and β1,6-glucans and a severe decrease of mannoproteins in the *Kloch1-1 *cells as compared to the wild-type counterparts. In addition, fine-structure determination of the β1,6-glucan polymer showed that, in the *Kloch1-1 *strain, the β1,6-glucans are shorter and have more branches than in the wild-type strain. Moreover, the *Kloch1-1 *cells showed a significantly improved capability of secreting heterologous proteins.

Recent reports provide evidences that defects in protein *N*-glycosylation can cause mitochondrial deficiencies. An ER-derived oxidative stress from misfolded proteins in an *erv29 *mutant of *S.cerevisiae *has been shown to led to UPR activation, which cause the accumulation of ROS from both the ER and mitochondria, and resulted in cell death [11]. It has also been reported that dosage attenuation of Dol-P- dependent GlcNAc-1-P transferase, *ALG7*, in haploid cells led to the loss of mtDNA and respiratory deficiency [12]. Also Eos1, a protein involved in the *N-*glycosylation, was required for tolerance to oxidative stress [13]. However, in most of the cases the mechanisms linking glycosylation and mitochondrial dysfunctions need to be elucidated. In this work we found that altered mitochondria functionality in cells deprived of the Golgi α-1,6-mannosyltransferase is caused by a defective calcium homeostasis.

## Results

### Altered mitochondrial biogenesis in Kloch1-1 cells

**Figure 1:**
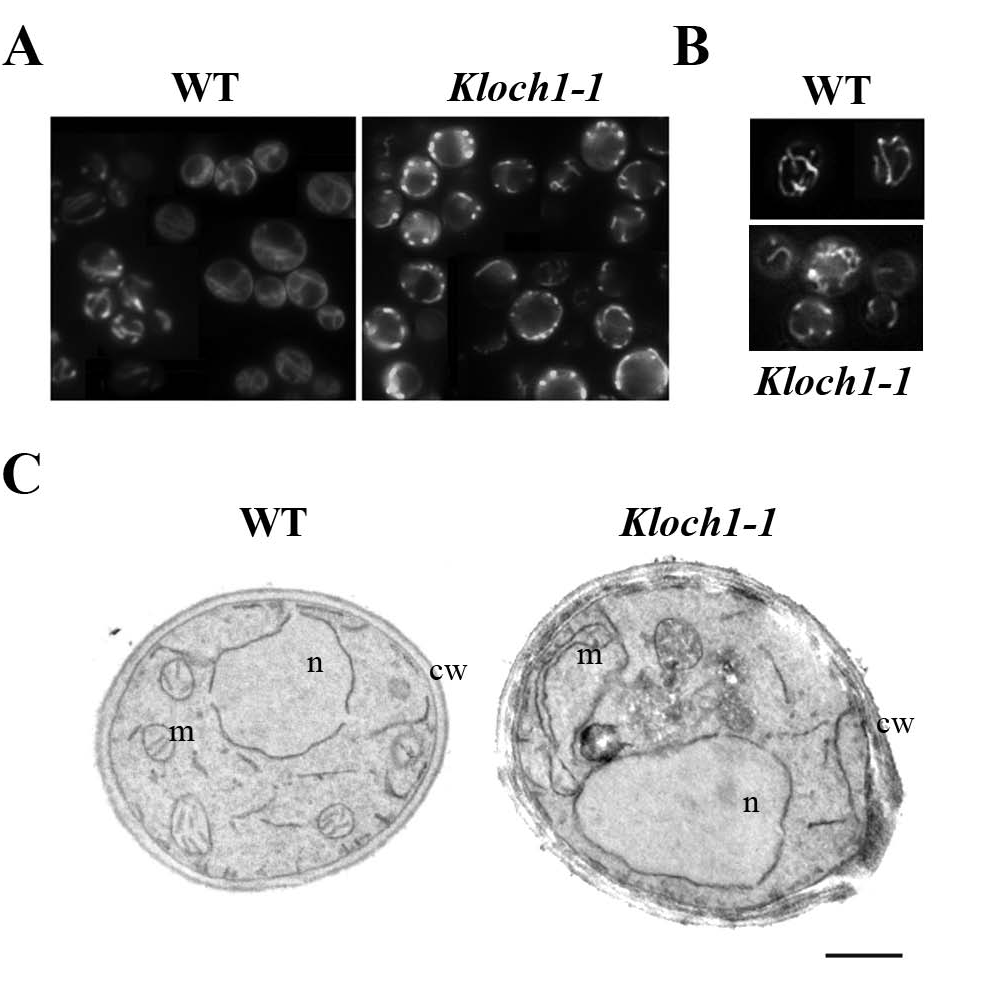
**Morphological and functional analysis of mitochondria in cells lacking α1,6-mannosyltransferase activity**. (A) DASPMI staining of *Kloch1-1 *cells and the parental strain. (B) GFP fluorescence of mitochondrial matrix of wt and mutant strains harbouring p426SD11 mtGFP vector. (C) Mitochondrial analysis by electron microscopy of *Kloch1-1 *cells and the parental strain. Cultures were grown to exponential phase into YPD medium. n, nucleus; m, mitochondrion; cw, cell wall; bar 2 μm.

The *K. lactis KlOCH1 *gene encodes the α-1,6-mannosyltransferase localized in the Golgi apparatus and involved in the glycosylation process and in cell wall morphogenesis. The *Kloch1-1 *mutant strain showed enhanced heterologous protein production [10]. However, the *Kloch1-1 *cells displayed a higher generation time in rich fermentable medium in comparison with wild type cells, and in the presence of respiratory carbon source, the biomass yield was strongly reduced. This prompted us to investigate if the lack of the α 1,6-mannosyltransferase activity in *K. lactis *cells could be associated with altered mitochondrial functionality. To this end cells were incubated with DASPMI, a fluorescent probe that is taken up by mitochondria as a function of membrane electrochemical potential. We found indeed a different distribution of the dye in the two strains: in the *Kloch1-1 *background the cells showed a punctuate pattern and some ring structures instead of the regular tubular network of the wild type counterpart (Figure 1A). In order to analyze possible alterations of the mitochondria structures, we transformed the mutant and wild type strains with a plasmid carrying the GFP fused to the mitochondrial signal sequence from the subunit 9 of the F_0_-ATPase from *Neurospora crassa*; this construct has been demonstrated to correctly deliver functional GFP into yeast mitochondria [14]. The fluorescence microscope observation of the mitochondrial matrix (Figure 1B) showed, for the mutant cells, an hyperbranched mitochondrial net not revealed by the DASPMI staining, whereas the mitochondrial morphology of the wild-type cells resulted identical to the one observed with DASPMI. To further study the altered mitochondrial morphology, the mutant strain and its isogenic wild type counterpart were analyzed by electron microscopy. In ultrathin sections of the wild type cells (Figure 1C) the mitochondria appeared as tubular structures, with normal morphology and typical cell peripheral distribution. The *Kloch1-1 *cells showed instead mitochondria that were either stretched and without crests in the middle part either round swollen at the ends, in agreement with the altered morphology observed by DASPMI staining.

**Figure 2:**
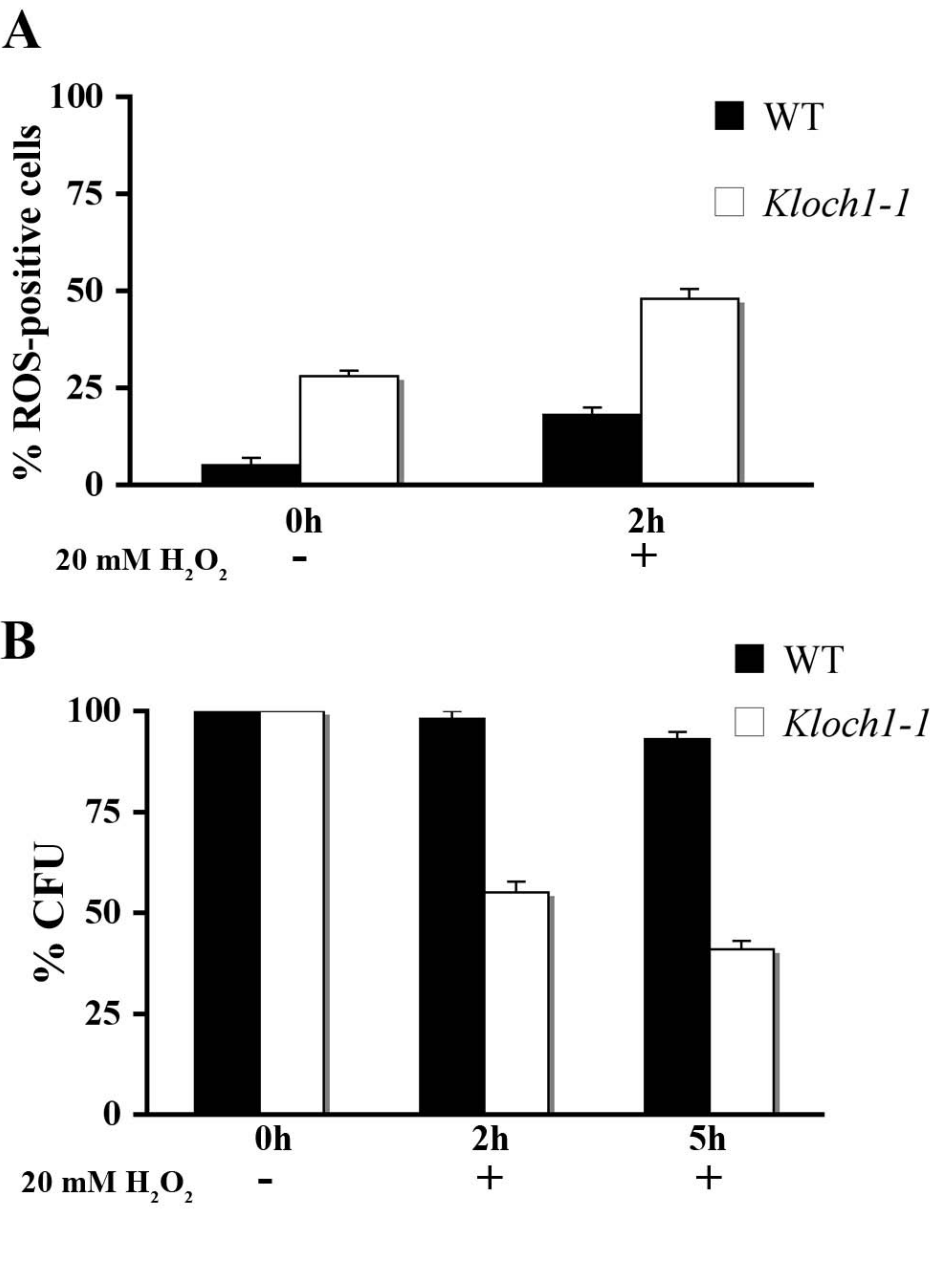
**Oxidative stress in *Kloch1-1 *cells**. (A) Estimation of ROS accumulation in the indicated strains by DHR-staining after growth to exponential phase in SD medium. Measurements were also obtained after exposure to H_2_O_2 _for 2 h. (B) Cell viability after H_2_O_2 _exposure: parental strain (black bars) and cells lacking α1,6-mannosyltransferase activity (white bars), grown to exponential phase on YPD medium, were challenged with 20 mM H_2_O_2 _for 2 or 5 h. The viability was evaluated plating the samples on YPD and was expressed as the CFU percentage of the corresponding untreated cultures. The values of both panels were the mean of three independent experiments and showed an SD < 10%.

**Figure 3:**
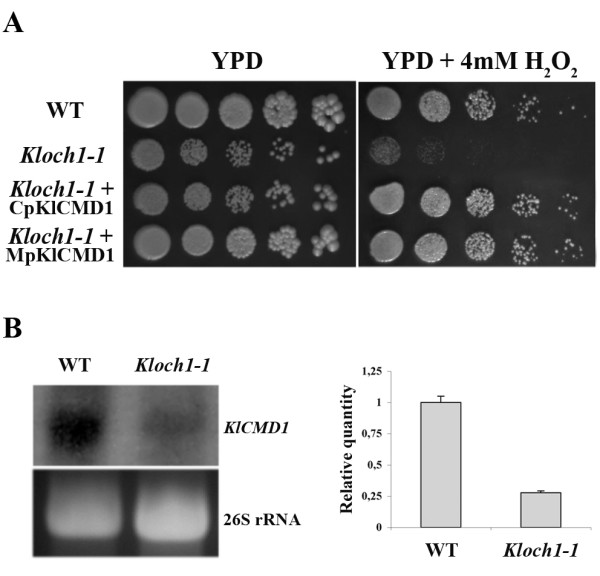
**Isolation of *KlCMD1 *as an extra-genic suppressor of H_2_O_2 _sensitivity**. (A) Genetic screen to identify suppressor(s) able to rescue the growth defect of *Kloch1-1 *cells. The *KlCMD1 *gene, responsible to allow again the growth of the mutant cells in medium containing the hydrogen peroxide was subcloned into centromeric and multicopy plasmids, CpKlCMD1 and MpKlCMD1 respectively. The growth at 28°C was monitored after 3 d; three independent transformants have been checked, obtaining identical results. (B) Comparison of transcript levels of calmodulin in parental and mutant strains by Northern blot analysis. RNAs were extracted from cells after growth for 48 h in SD minimal medium. The same amount of total RNA (40 μg) from the strains was loaded on each lane; the ethidium bromide-stained gel of the autoradiogram is shown in the bottom part of the panel and the mRNA loading was normalized using the 26S rRNA bands. Quantification, by densitometric analysis, of the radiolabeled signal on the blot is shown in the right part of the panel. The hybridization signal for wild type strain was set as 1.

Mitochondrial dysfunction is often associated with accumulation of reactive oxygen species (ROS), we therefore evaluated the amount of ROS by using the fluorescent dye 123 dihydrorhodamine (DHR). This compound accumulates inside the cells and it is oxidized to the corresponding fluorescent chromophore by ROS. The fluorescence microscope observation revealed that 28% of *Kloch1-1 *cells accumulated ROS as compared to 5% of the parental strain; these values increased up to 48% and 18% in the mutant and wild type cells respectively after challenge with a generator of oxidative stress, such as the hydrogen peroxide (Figure 2A). To further investigate the oxidative stress taking place in the mutant, *K. lactis *cells were challenged for 2 and 5 h with a cytotoxic concentration of 20 mM hydrogen peroxide. A strong reduction of the survival rate was observed after a 2 hours treatment in cells lacking the α 1,6-mannosyltransferase activity (Figure 2B), and after 5 hours only 41% of mutant cells survived versus 93% of the parental ones. In addition, the mutant strain was not able to grow in the presence of 4 mM hydrogen peroxide (Figure 3A).

### Isolation of KlCMD1 as extragenic suppressor of oxidative stress occurring in Kloch1-1 cells

In order to highlight genetic interactions underlying the phenotypes observed, we performed a screen to identify multicopy suppressors able to relieve the growth defect of cells lacking α 1,6-mannosyltransferase activity on YPD supplemented with 4 mM hydrogen peroxide (Figure 3A). Three of the plasmids, isolated from the corresponding clones that survived the selection procedure, resulted to be identical and were further analyzed. Sequencing analysis revealed that the *K. lactis *DNA fragment present in the plasmid contained an ORF of 441 bp with 1000 bp upstream of the putative ATG start codon and 100 bp downstream of the putative stop codon. The protein encoded by this ORF resulted to be *KlCMD1*, the homologue of the *S. cerevisiae *calmodulin gene [15]. The gene was able to restore the growth defect of *Kloch1-1 *cells on 4 mM H_2_O_2 _also when cloned in a centromeric plasmid (CpKlCMD1).

We wondered if transcriptional variations of *KlCMD1 *gene could occur in *Kloch1-1 *mutant cells as compared to the parental strain. In these cells we effectively observed a strong reduction of the mRNA of the calmodulin protein as revealed by northern blotting analysis (Figure 3B).

**Figure 4:**
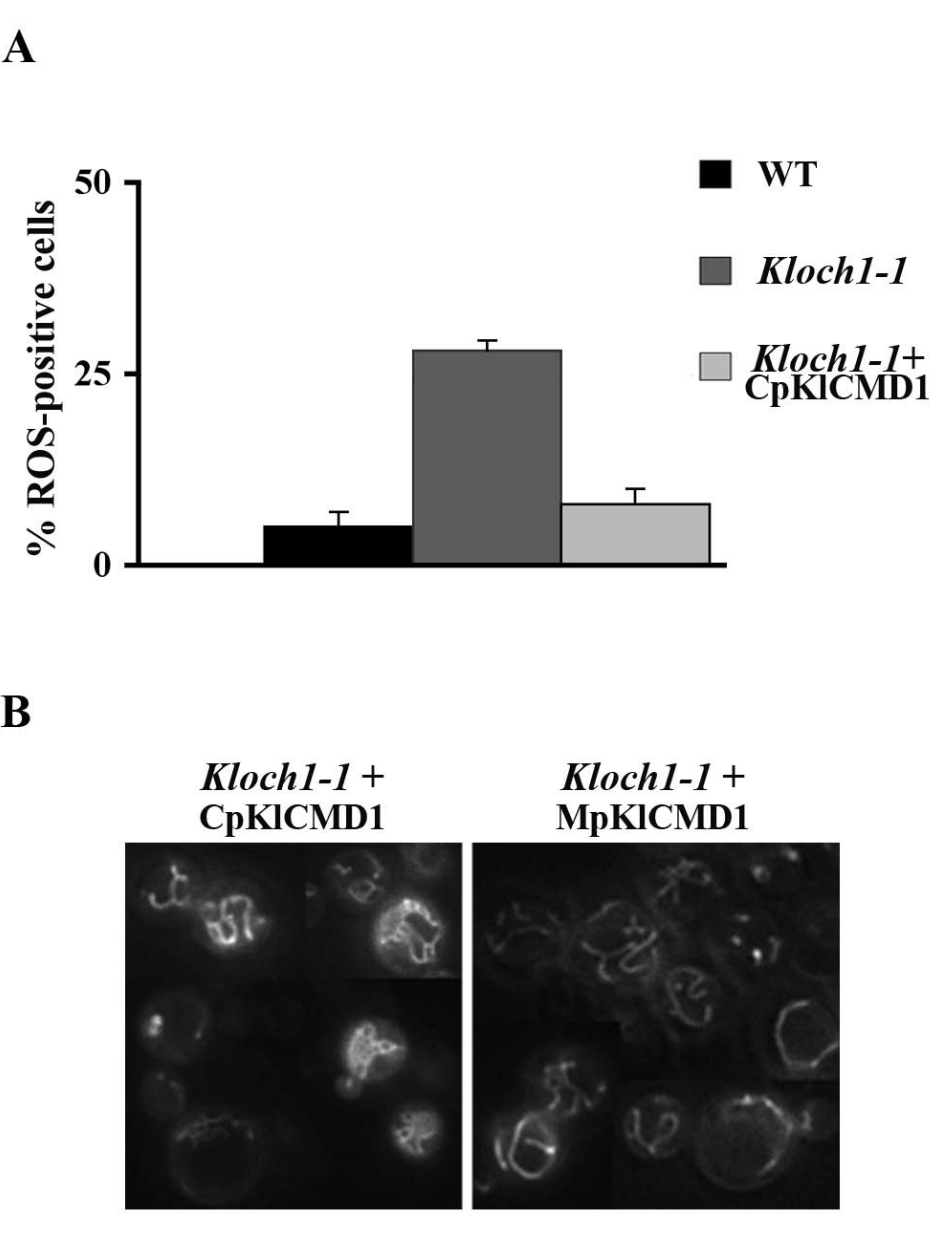
**Functional mitochondrial analysis of *Kloch1-1 *cells transformed with CpKlCMD1 plasmid**. (A) Estimation of ROS accumulation in WT cells (black bar) and *Kloch1-1 *mutant strain harbouring empty (dark grey bar) or CpKlCMD1 (light grey bar) vectors by DHR-staining after growth to exponential phase in SD medium. The values were the mean of three independent experiments and showed an SD < 10%. (B) DASPMI staining of mutant cells transformed with centromeric (left side of the panel) or multicopy (right side of the panel) plasmids containing *KlCMD1 *gene.

The presence of *KlCMD1 *on a centromeric plasmid in the mutant strain was able to reduce the oxidative stress of these cells as revealed by the DHR staining. The amount of positively stained cells of the transformants, in fact, was significantly reduced in comparison to that of the mutant cells: 8% *versus *28% respectively (Figure 4A). However, the *Kloch1-1 *cells transformed with CpKlCMD1 did not show an increase in the survival capabilities with respect to the mutant cells, when challenged for 2 hours with 20 mM (additional file 1).

The mitochondrial functionality of the *Kloch1-1 *cells carrying either the calmodulin on a centromeric (CpKlCMD1) or on a multicopy plasmid (MpKlCMD1) was studied by DASPMI staining (Figure 4B). The *Kloch1-*1 cells transformed with CpKlCMD1 showed a functional hyperbranched mitochondrial net in contrast to the dots phenotype, typical of the mutant (see Figure 1A). These data indicate that the mitochondrial net that was visualised by mitoGFP in *Kloch1-1 *cells (see Figure 1B) became able to establish a membrane electrochemical potential just by adding a few copies of calmodulin in these cells.

In addition, when the mutant cells were transformed with MpKlCMD1, the DASPMI staining revealed a wild type-like mitochondrial network, even if in some cases ring structures were still present (Figure 4B).

**Figure 5:**
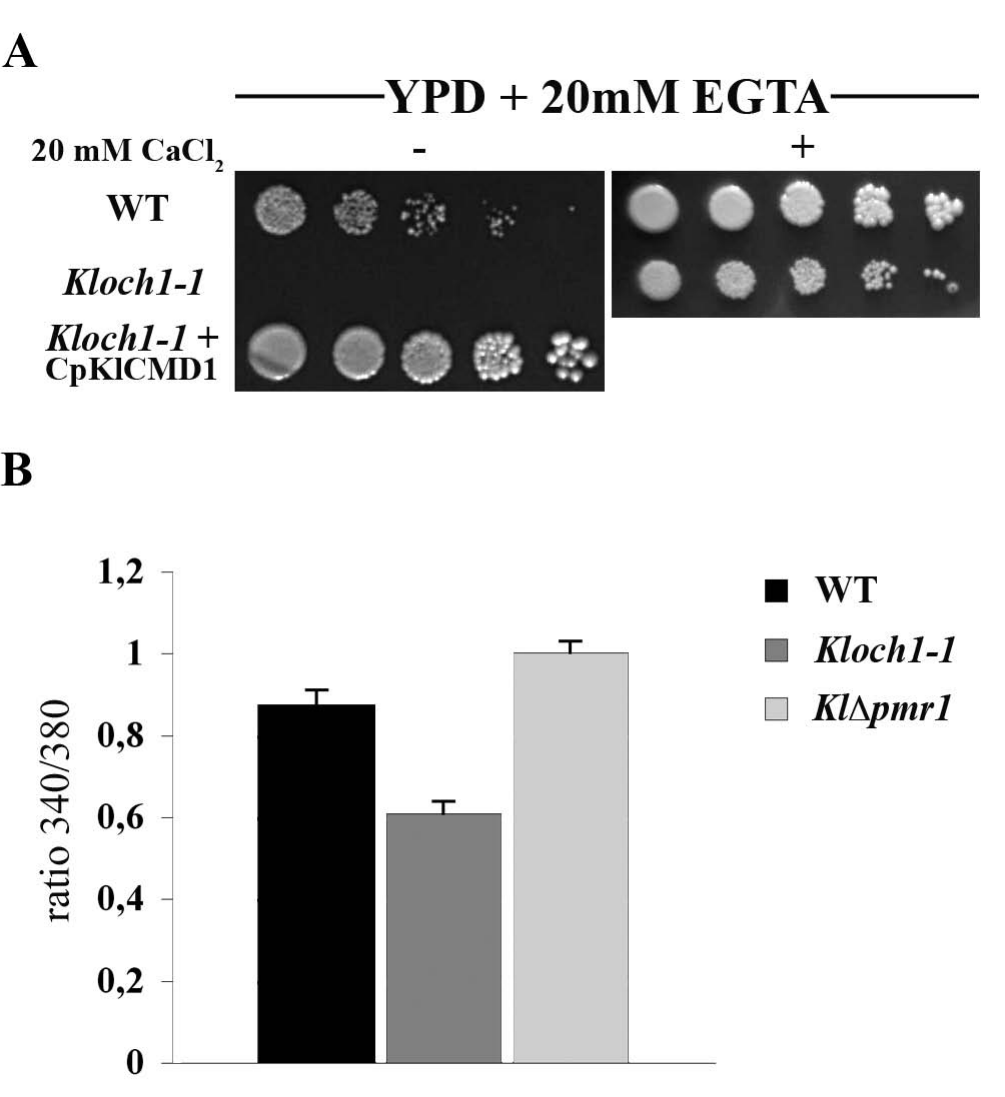
**Altered calcium homeostasis in *Kloch1-1 *mutant**. (A) Growth of indicated yeast strains in the presence of 20 mM EGTA. YPD-EGTA plates with and without 20 mM CaCl_2 _were spotted with 5 μl of 10-fold serial dilutions of cells from exponential cultures and growth at 28°C was monitored after 3 d; three independent transformants have been checked, obtaining identical results. (B) Intracellular calcium content in WT (black bar) and *Kloch1-1 *(dark grey bar) cells measured with FURA-2 AM, expressed as the ratio of fluorescence excitation intensities (340/380 nm). Ca^2+ ^ion measurements of *Klpmr1*Δ strain (light grey bar) was also reported as a control.

In addition to the generation of cellular energy, mitochondria also play an important role in regulating calcium homeostasis [16]. Based on this and on the suppression of the mutant phenotypes by calmodulin, we looked for possible calcium homeostasis alterations in *Kloch1-1*cells. We thus investigated if cells lacking α 1,6-mannosyltransferase activity could grow in presence of EGTA, a cationic chelator. As reported in the panel A of the figure 5, the growth of mutant cells was strongly inhibited when YPD plates were supplemented with this chelating agent. This inhibition was suppressed by adding in the growth medium, 20 mM CaCl_2 _together with the EGTA, indicating that an altered calcium homeostasis occurs in the mutant strain.

Intracellular calcium determinations, carried out using Fura-2AM, indeed showed that the calcium content of *Kloch1-1 *cells was significantly reduced with respect to the cation concentration present in the wild-type cells determined in the same conditions (Figure 5B). Measurements employing cells deleted for the Golgi Ca^2+^-ATPase, KlPmr1p, previously reported to have increased cytosolic calcium content, were used as additional calibration control.

### Increased calcineurin activity is required in Kloch1-1 cells for normal mitochondrial morphology and cell wall structure

Since calmodulin acts as a mediator of calcium signals in eukaryotic cells mainly through the Ca^2+^/calmodulin-dependent phosphatase calcineurin, we investigated the possible role of this phosphatase in the mitochondrial phenotypes of *Kloch1-1 *mutant cells.

**Figure 6:**
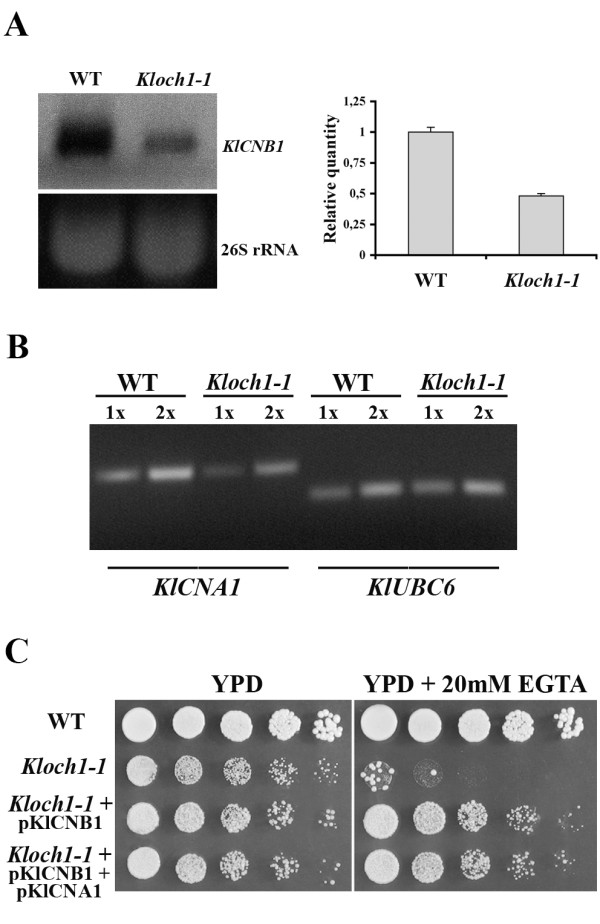
**Analysis of calcineurin in *Kloch1-1 *cells**. (A) Northern Blotting analysis of *KlCNB1 *WT and *Kloch1-1 *cells. The same amount of total RNA (40 μg) from the strains was loaded on each lane; the ethidium bromide-stained gel of the autoradiogram is shown in the bottom part of the panel and the mRNA loading was normalized using the 26S rRNA bands. Quantification, by densitometric analysis, of the radiolabeled signal on the blot is shown in the right part of the panel. The hybridization signal for wild type strain was set as 1. (B) RT-PCR semi-quantitative analysis of *KlCNA1 *gene. Exponentially growing wild-type and *Kloch1-1 *cells were collected and RNA was isolated. Reverse transcription and PCR reactions with the specific primers described in materials and methods was performed. Shown is the electrophoresis in a 2% agarose gel of 10 μl of each PCR reaction with 5 μl (1×) and 10 μl (2×) of cDNA as template. RT-PCR of the *KlUBC6 *gene was performed as an internal control. RNA extraction was performed twice and the results shown are representative of four independent RT-PCR experiments. (C) Growth of indicated yeast strains onto solid medium supplemented with 20 mM EGTA.

Calcineurin requires both the regulatory and the catalytic subunits for full activity, and the *KlCNB1 *and *KlCNA1 *genes respectively were isolated (see Materials and Methods). The reduced expression of *KlCMD1 *observed in the mutant strain prompted us to first analyze whether a similar behaviour could be observed also for the calcineurin subunits. The *Kloch1-1 *cells effectively showed a decrease of *KlCNB1 *transcript in comparison to the wild type cells, and a similar result was observed also for the catalytic subunit (Figure 6A and 6B). In order to obtain the expressional balance of both regulatory and catalytic subunits of calcineurin, we transformed *Kloch1-1 *strain with the plasmids harbouring *KlCNB1 *(pKlCNB1) and *KlCNA1 *(pKlCNA1) genes. Surprisingly, the effect of the increased dosage of the regulatory subunit alone in the *Kloch1-1 *cells on the EGTA sensitivity of the mutant strain was identical to that of the overexpression of both subunits (Figure 6C). Similar results were also obtained when the amount of ROS was analyzed: 15% of the *Kloch1-1 *cells transformed with pKlCNB1 alone or together with pKlCNA1 were positive to the staining with DHR as compared to 28% of the mutant cells. In this case the suppression was not complete as compared to the almost complete recovery observed in *Kloch1-1 *cells by increasing the dosage of calmodulin gene.

**Figure 7:**
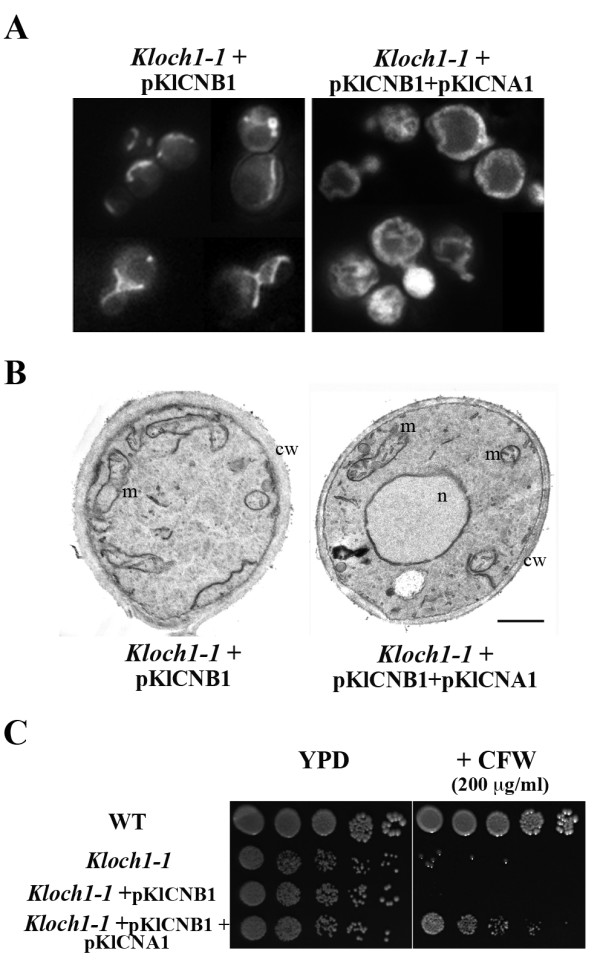
**Phenotypical analysis of *Kloch1-1 *overexpressing *KlCNB1 *or both subunits of calcineurin**. (A) DASPMI staining of the *Kloch1-1 *cells harbouring pKlCNB1 plasmid alone or with the pKlCNA1 plasmid. (B) Ultra-thin sections of above strains. n, nucleus; m, mitochondrion; cw, cell wall; bar 2 μm. (C) Serial dilutions of cultures from the same strains onto YPD agar plates supplemented with the cell wall interfering agent calcofluor white (CFW). The growth at 28°C was monitored after 3 d; three independent transformants have been checked, obtaining identical results.

We then looked up to the mitochondrial structures and functionality by DASPMI staining of the transformants. Mutant cells carrying pKlCNB1 vector alone showed a partial recovery of the tubular phenotype. We observed a reduced amount of dots and the appearance of tubules, whereas the same cells transformed also with the pKlCNA1 plasmid showed the typical tubular wild type-like network, although with a peripheral distribution (Figure 7A). By electron microscopy analysis we observed only a partial recovery of tubular mitochondria in *Kloch1-1 *cells harbouring only the pKlCNB1 vector (Figure 7B). However, when in the same strain we co-overexpressed also the catalytic subunit of calcineurin, the relieve was almost complete: mitochondria appeared very similar to the wild type ones and mitochondrial cristae were visible, in agreement with the structures observed by fluorescence microscopy (Figure 7B).

We previously reported that the *Kloch1-1 *mutant had increased cell wall thickness and some dark-stained rims were present within the amorphous layer in comparison with wild-type cells [10]. Notably, in cells depleted of α1,6 mannosyltransferase activity but transformed with the pKlCNB1 and pKlCNA1 plasmids, the thickness of the cell wall resembled that of the wild-type parent (Figure 7B). In agreement with the electron microscopy observations, we found that the ability of the mutant cells to grow in presence of the cell wall-perturbing agent calcofluor white was completely restored only when the cells were transformed with both subunits of calcineurin (Figure 7C). Identical results were also obtained when congo red, another molecule interfering with the cell wall, was used (data not shown). This phenotype has to be ascribed to the increased activation of the calcineurin signaling pathway that generates a rescue of a normal biogenesis of cell wall.

## Discussion

Protein *N*-glycosylation is one of the fundamental metabolic pathways in cell fate. Deciphering how *N*-glycosylation controls specific metabolic and signaling events has become important to unraveling the underlying basis of cellular behavior. In yeasts, outer chain branching is initiated in the Golgi apparatus by the α-1,6-mannosyltransferase Och1p. Here, we reported that altered mitochondrial functionality and oxidative stress take place in *K. lactis *cells carrying a mutation in *KlOCH1 *gene. The mutant cells showed also a reduction in the calcium content and in the expression of genes related to calcium signalling.

Although a direct link between *N*-glycosylation defects and mitochondrial functionality has not been reported, underglycosylation of proteins destined for the mitochondria could interfere with, or abolish, the import of these proteins into the organelle. Indeed, a 45 kDa *N*-glycoprotein has been identified in rat liver inner mitochondrial membranes that physically interacts with complex I and the F_1_F_0_-ATP synthase [17]; Tim11p, its yeast homologue, has one potential *N*-glycosylation site. Also, mutations in the signal recognition particle (SRP) receptor have been shown to disrupt the reticular structure of both the ER and mitochondria in yeast [18], suggesting that a proper ER structure and/or functionality is required for maintaining the mitochondrial network. It is conceivable that the underglycosylation of proteins has adverse effects on the early secretory compartments structure and functionality, which, in turn, influences mitochondria characteristics.

Moreover, we found that *KlCMD1 *gene was a suppressor in *Kloch1-1 *cells of either ROS accumulation or sensitivity to the oxidative stress, when H_2_O_2 _was added in the growth medium. On the other hand, the increased dosage of this Ca^2+^-signalling gene was not able to increase the survival rate of the mutant cells undergoing a treatment with cytotoxic concentration of the same oxidant agent. These data suggest that, in the mutant cells, the altered defence mechanisms against high concentration of a ROS generator were not fixed by calmodulin itself.

The *Kloch1-1 *cells also showed a reduction in the calcium content, accompanied by a reduction in the expression of the *KlCMD1*, *KlCNA1 *and *KlCNB1 *genes, encoding for key components of the calmodulin/calcineurin signalling pathway. In fungi, conserved signal transduction pathways control fundamental aspects of growth, development and reproduction. Two important classes of fungal signalling pathways are the mitogen-activated protein kinase (MAPK) cascades and the calcium-calcineurin pathway. They are triggered by an array of stimuli and target a broad range of downstream effectors such as transcription factors, cytoskeletal proteins, protein kinases and other enzymes, thereby regulating processes such reproduction, morphogenesis and stress response [19,20]. We can thus hypothesize a possible activation of a MAPK signaling in *Kloch1-1 *cells that in turn could down modulate the calcium signaling pathway. It has been found that in *K. lactis *cells deleted in *PMR1 *gene and sharing phenotypes with the *Kloch1-1 *mutant, such as cell wall defects, oxidative stress and altered calcium homeostasis, the HOG1 MAPK cascade resulted activated [21].

*S. cerevisiae *calmodulin, a Ca^2+ ^binding protein, regulates many cell processes both depending or not upon the intervention of Ca^2+ ^ions. Among those Ca^2+^-dependent is the organization of the actin cytoskeleton; moreover mutations in *CMD1 *resulted colethal, suggestive of functional interactions, with the inactivation of genes encoding components of the glycosylation pathways like ANP1, CWH8 and MNN10 [22]; mutations in such genes also result in altered morphology of actin cytoskeleton. We should also take into account that, although *och1 *deletion mutant of *S.cerevisae *in the BY4741 background was sensitive to EGTA, the mutant strain was not altered in the expression of calmodulin and calcineurin genes (Zanni *et al*., unpublished results). However, we can not exclude the possibility that a reduction in the activity of the calcium signalling proteins can occur in the *OCH1 *deleted cells.

Mitochondrial plasticity and functionality strongly depend upon the interactions between mitochondria and cytoskeleton. Several shape-related proteins have been described in S.cerevisiae, localized on the mitochondria surface and reported to interact with actin [23,24]; however the individual role and underlaying mechanisms are still unsolved.

Another unanswered question is how do cells change the mitochondrial shape upon cell signals. In the case of calcium signalling, a relevant player could well be Gem1p, a member of the Miro GTPase family [25]; Gem1p is also localized on the outer mitochondrial membrane with its GTPase domain and, most notably, its EF-hand calcium binding domain exposed in the cytosol.

We are tempting to speculate that the altered calcium availability we observed in *Kloch1-1 *cells could be originated by a defective calcium membrane channel Mid1/Cch1. In fact, it has been demonstrated that *S. cerevisiae *Mid1 requires a full glycosylation to correctly localize and assemble at the level of the plasma membrane [26].

In mammalian cells Ca^2+ ^influx through voltage-dependent Ca^2+ ^channels (VDCCs) causes a rapid halt in mitochondrial movement and induces mitochondrial fission. VDCC-associated Ca^2+ ^signaling stimulates phosphorylation of dynamin-related protein 1 (Drp1) at serine 600 via activation of Ca^2+^/calmodulin-dependent protein kinase Iα (CaMKIα). In neurons and HeLa cells, phosphorylation of Drp1 at serine 600 and dephosphorylation at serine 637, both calcineurin-dependent, are associated with increase in Drp1 translocation to mitochondria [27,28].

Nevertheless, one cannot exclude that defective *KlOCH1 *gene induce reduced glycosylation of other proteins relevant for calcium handling; scrutiny of such picture will deserve future work.

## Conclusions

A proper functioning of outer chain-extension of mannoproteins in *K. lactis *is required for correct calcium homeostasis. The impairment of *KlOCH1 *results in a low calcium/calmodulin based signaling and altered mitochondria morphology and functionality. The reported data strongly indicate a novel link between relevant cell processes taking place in separate compartments.

## Methods

### Yeast Strains and Growth Conditions

The strains used in this study were MW278-20C (MAT a, *ade2*, *leu2*, *uraA*), CPV3 (MAT a, *ade2*, *leu2*, *uraA, Kloch1-1*) and CPK1 **(**MAT a, *ade2, leu2, uraA*, *KlPMR1::Kan *R). Yeast strains were grown in YPD medium (1% yeast extract, 1% peptone, 2% glucose) or SD minimal medium (2% glucose, 0.67% yeast nitrogen base without amino acids) with the appropriate auxotrophic requirements. Fivefold serial dilution from concentrated suspensions of exponentially growing cells (5 × 10^6 ^cell/ml) were spotted onto synthetic YPD agar plates supplemented or not with 4 mM H_2_O_2_, 20 mM EGTA, 200 μg/ml Congo red or 50 μg/ml CFW and the plates were incubated at 30°C for 48 h.

### Plasmids Construction

Construction of the pCXJ3-U and pCXJ6-L plasmids: the kanamycin-resistance encoding gene (*kan*) was excised by PstI digestion from pCXJ3 and pCXJ6 [29] plasmids, giving muticopy vectors with the selectable marker URA3 or LEU2, respectively.

Construction of the pCXJ3-K plasmid: the URA3 gene was excised by BglII digestion from pCXJ3 plasmid, obtaining muticopy vector with the selectable marker KAN.

Construction of the CpKlCMD1 and MpKlCMD1 plasmids: the 1545 bp fragment containing the full ORF (444 bp) of *KlCMD1 *plus 1000 bp upstream and 100 bp downstream was amplified by PCR, using primers modified with the recognition site for the restriction endonuclease BamHI. The PCR fragment encoding the KlCmd1p was cloned into the pGEM-T-Easy vector (Promega) according to the manufacturer's instructions, giving pGEM-KlCMD1 and the gene correctness was confirmed by DNA sequencing (MWG Biotech, Martinsried, Germany). The fragment was excised by BamHI digestion from pGEM-KlCMD1 and was ligated into the centromeric (pCXJ20) or multicopy (pCXJ6-L) vectors, linearized by the same endonuclease, to obtain the CpKlCMD1 and MpKlCMD1 plasmids respectively.

Construction of the pKlCNB1 and pKlCNA1 plasmids: the *KlCNB1 *and *KlCNA1 *genes were PCR amplified from *K. lactis *DNA genome using the primers 5'-CG**GGATCC**GGGCAGAGAGCAGGTTCAAC-3' and 5'-CG**GGATCC**GCTGCTTCACATTCATACGCGC-3', 5'-CG**GGATCC**CGTCAGCCCCAGCTTCCTCATC-3' and 5'-CG**GGATCC**CCGGTGCCGTTGTTGACAAGGG-3' respectively (the BamHI restriction site is underlined). The PCR products were ligated into the pGEM-T-Easy vector (Promega) giving the pGEM-KlCNB1 and pGEM-KlCNA1 plasmids.

After sequencing (MWG Biotech, Ebersberg, Germany) the fragments were successively cloned in BamHI-digested pCXJ3-U and pCXJ3-K plasmids, obtaining pKlCNB1 and pKlCNA1 vectors, respectively.

### Yeast Transformation and Selection of Suppressor Genes

The *Kloch1-1 *strain was transformed to saturation with the yeast genomic library constructed in the pKep6 multicopy vector (kindly provided by Wesolowsky-Louvel) by electroporation [30]. All the Ura^+ ^transformants were replicated on to YPD medium supplemented with 4 mM H_2_O_2_. The plasmids isolated from the Ura^+^/H_2_O_2_^R ^transformants were used to transform the *Kloch1-1 *strain. Plasmids capable of restoring the H_2_O_2_^R ^phenotype to the *Kloch1-1 *after retransformation were analyzed. Restriction enzymes analysis of the genomic fragments from the isolated plasmids showed that one of these plasmids carrying an insert of about 8000 bp was able to restore the H_2_O_2_^R ^phenotype. The plasmid was then sequenced (MWG Biotech). The 1600 bp fragment contained the full ORF (444 bp) of *KlCMD1 *plus 1000 bp upstream and 100 bp downstream.

### Stress Condition and Viability

Yeast cells were grown aerobically at 28°C in liquid medium for 24 h and were challenged with hydrogen peroxide. This was directly added to the growth medium to the final concentration of 20 mM. Untreated cultures were incubated in parallel over the same periods. Viability was determined by colony counts on YPD plates after 2 and 5 h of incubation at 28°C and was expressed as the percentage of the corresponding control cultures. The values are the mean of three independent experiments with a SD < 15%.

### Measurement of Intracellular Oxidation Levels

The oxidant-sensitive probe dihydrorhodamine 123 (Sigma) was used to measure intracellular oxidation levels in yeast according to [31].

Cells were concentrated by centrifugation and resuspended in 10 μl of fresh medium. Five microliters of cells were loaded onto slides and observed immediately under epifluorescence microscopy (excitation at 488 nm and emission at 530 nm). At least 300 cells per sample were scored manually as fluorescent or nonfluorescent.

### Fluorescence Microscopy

Cells grown in 2% glucose medium were harvested in exponential growth phase (6 × 10^7 ^cells/ml), washed with water and then incubated for 30 min in the presence of 5 μM of DASPMI [32]. Epifluorescence microscopy was carried out with a Zeiss Axiophot microscope fitted with a 100× immersion objective and a standard FITC filter set.

### Electron Microscopy

Cells were prepared as described in [21].

### Northern Blot Analysis

Total RNA of *K. lactis *strains was extracted by the hot phenol method [33]. The RNAs were quantified by absorption (OD_260_) and separated by denaturing agarose electrophoresis. After electrophoresis the RNAs were transferred to nylon membranes and hybridized with 32P-labeled random primed probes (Roche, Lewes, East Sussex, United Kingdom). All the probes were PCR amplified from the *K. lactis *DNA genome. The 1300-bp PCR product of *KlCMD1 *was obtained using primers 5'-CGGGATCCCGTACCCTGATAGCTCTACC-3' and 5'-CGGGATCCGTGCGTAATTTGAGCGATGG-3'. The fragment of 410 bp containing the *KlCNB1 *gene was obtained with primers 5'-GAATTGAAATGGGAGCAGCA-3' and 5'-CTTGAAAATCAACATCTCCGC-3'. The densitometric analysis was done with an image analyzer (Phoretix 1D; Non Linear Dynamics Ltd.).

### Semi quantitative RT-PCR

Total RNA extracted as before was subjected to TURBO™ DNase treatment according to manufacture's instructions (Ambion, USA). Reverse transcription was performed using Promega Reverse Transcription System with 1 μg total RNA to yield 20 μl cDNA. PCRs were then performed to determine the linear range of amplification for the genes that would allow a semi-quantitative assessment of expression levels. The primers used for *KlCNA1 *were 5'-GTTAATGCAGCTCTGCGAGTC-3' and 5'-CACGTGATAGTCGTCCTTCT-3', while for *KlUBC6 *were 5'-ATTACGTGATTACCGGTCCA-3' and 5'-GCCTCTGGATGATAATCACT-3'. The optimal parameters determined for each PCR were 95°C, 30 s; 52°C, 30 s; 72°C, 30 s; and 20 cycles for both the *KlCNA1 *and *KlUBC6 *genes. The primers used were designed to yield small amplicons (*KlCNA1*, 241 bp; *KlUBC6*, 178 bp;) to improve the efficiency and reproducibility of the PCR. 10 μl of each PCR reaction with 5 μl (1×) and 10 μl (2×) of cDNA diluted 1:5 as template were separated on a 2% agarose gel, stained with ethidium bromide and photographed.

### Ca^2+ ^Measurements

A suspension of 50 μl freshly prepared spheroplasts were diluted in 1 ml of spheroplast buffer (SB) containing 1 M sorbitol, 50 mM Tris buffer, pH 7.5, 10 mM Mg^2+ ^and left into a polylysine-coated plate in agitation at 4°C overnight. Plates were washed twice with 1 ml of SB. Spheroplasts were incubated for 60 min at 37°C in standard reaction medium (125 mM sucrose, 65 mM KCl, 10 mM HEPES, pH 7.2 and 500 μM ethanol) in the presence of 0.1 mg/ml bovine serum albumin (Sigma) and 10 μM Fura-2AM (Molecular Probes, Eugene, OR). To measure fluorescence changes, a Hamamatsu Argus 50 computerized analysis system was used, recording every 6 sec the ratio between the values of light intensity at 340 and 380 nm stimulation. Spheroplasts were prepared according to [32].

## Authors' contributions

EZ, DU, designed, carried out the experiments, analyzed data and drafted the manuscript. FF performed the genetic screening, analyzed data and commented on the manuscript. CF performed the calcium measurements, analyzed data and commented on the manuscript. AR participated to the electron microscope analysis. PM performed the electron microscope analysis, analyzed data and commented on the manuscript. CP designed experiments, analyzed data and commented on the manuscript. DU supervised the project and corrected the final manuscript. All authors read and approved the final manuscript.

## Supplementary Material

Additional file 1 **Cell viability after H_2_O_2 _challenge**. Indicated strains, grown to exponential phase on YPD medium, were challenged with 20 mM H_2_O_2 _for 2 h. The viability was evaluated plating the samples on YPD and was expressed as the CFU percentage of the corresponding untreated cultures. The values were the mean of three independent experiments and showed an SD < 10%. Click here for file
