# Supplementary figures and images for: The Golgi α-1,6 mannosyltransferase KlOch1p of Kluyveromyces lactis is required for Ca2+/calmodulin-based signaling and for proper mitochondrial functionality
Source: BMC Cell Biol. 2009 Dec 14;10:86. doi: 10.1186/1471-2121-10-86 (PMC2797761; doi:10.1186/1471-2121-10-86)

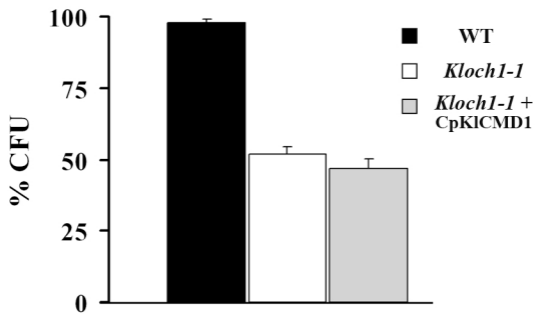

Supplement: Additional file 1 — Cell viability after H2O2 challenge. Indicated strains, grown to exponential phase on YPD medium, were challenged with 20 mM H2O2 for 2 h. The viability was evaluated plating the samples on YPD and was expressed as the CFU percentage of the corresponding untreated cultures. The values were the mean of three independent experiments and showed an SD < 10%. [file 1471-2121-10-86-S1.PDF]
